# The Psychosocial Impact of Indwelling Pleural Catheters: A Scoping Review

**DOI:** 10.7759/cureus.41689

**Published:** 2023-07-11

**Authors:** Adam M Peel, Eleanor K Mishra

**Affiliations:** 1 Community Research, Norfolk Community Health and Care NHS Trust, Norwich, GBR; 2 School of Health Sciences, University of East Anglia, Norwich, GBR; 3 Norwich Medical School, University of East Anglia, Norwich, GBR

**Keywords:** scoping review, psycho-social impact, systematic scoping review, quality of life (qol), tunneled pleural catheter, psychosocial impact, pleural effusion, ipc, indwelling pleural catheter

## Abstract

We aimed to identify research on the psychosocial impact of Indwelling Pleural Catheters (IPC); report on the extent, range, and nature of studies; and summarize the findings. A secondary aim was to capture reports on patient support needs and/or self-management of IPC. A systematic literature search was undertaken, with evidence synthesis planned if sufficient literature was identified. We searched ten databases available through the United Kingdom National Health Service Knowledge and Library Hub: the British Nursing Index (BNI), Cumulative Index to Nursing and Allied Health Literature (CINAHL), Cochrane, Excerpta Medica Database (Embase), Exerpta Medica Care (Emcare), E-thesis Online Service (EThOS), Medical Literature Analysis and Retrieval System Online (Medline), National Grey Literature Collection, Psychological Information Database (PsycInfo), and PubMed. We included studies reporting on the psychosocial impact of indwelling pleural catheters or their effect on quality of life (QoL). The latter was limited to those studies using qualitative research methods from which we could identify psychosocial impacts. The evaluation of psychosocial factors was not the primary objective of any identified study, and we found no studies in which quality of life was assessed using qualitative methods. Two studies met the inclusion criteria but only tangentially. While indwelling pleural catheters may improve the quality of life in patients with pulmonary effusion when assessed quantitatively, there is a dearth of research examining their psychosocial impact.

## Introduction and background

Pleural effusion - an abnormal collection of fluid between the layers of the pleura - can cause debilitating breathlessness and chest pain. Although there are a number of possible causes, they are a common complication of advanced malignancy (both thoracic and extra-thoracic) [1]. It is estimated that between 200,000 and 250,000 pleural effusions occur in the United Kingdom (UK) each year [2], and as many as 15% of all patients diagnosed with malignancy may experience an effusion. The incidence of pleural disease [3] and the prevalence of cancer may be rising; these two factors combined suggest the incidence of malignant pleural effusion (MPE) is also likely to increase [4].

Malignant effusions are associated with a high mortality rate and short survival time as they tend to signify advanced or metastatic disease [5]. Breathlessness as a result of recurrent MPE is one of the main factors decreasing the quality of life (QoL) in patients with cancer [6]. Treatment for such effusions is primarily the palliation of symptoms through fluid drainage. For recurring effusions, it is increasingly common to use an indwelling pleural catheter (IPC); a silicone tube tunnelled under the skin and into the pleural cavity. This remains in situ and permits the regular drainage of fluid as required. The first commercially available, purpose-made catheter system was approved (for use in the relief of dyspnoea in patients with MPE) by the United States (US) Food and Drug Administration in 1997. However, the adaptation of other medical catheter systems for this purpose was reported as early as 1994 [7].

The use of IPC is in line with the UK government’s ambition to deliver more services in the community, close to - or in - the home [8]. IPC insertion is typically undertaken as a day procedure, with subsequent drainage and re-dressing of the IPC managed in the community by the patient, carer, or a community nursing team. Drainage may be required several times a week; for some patients, IPC may remain in place for months to a year or more.

The alternatives to IPC include repeated thoracenteses, chest drains, and pleurodesis (for example, by talc poudrage). Research in the last decade has evaluated these competing management strategies, and both chest drain and pleurodesis and IPC implantation have been shown to improve dyspnoea and quality of life scores [9]. Draft clinical guidelines [10] state that the relative risks and benefits of both should be discussed with patients and that good practice includes consideration of ‘the psychological implications and potential altered body image aspects of having a semi-permanent tube drain in situ. While studies of IPC efficacy have captured their impact on dyspnoea and used validated questionnaires to assess the quality of life [11], studies examining the psychosocial impact of IPC insertion and management have not - to the best of our knowledge - been published, and the frequency of qualitative data capture (and its reporting) in the published studies of medical efficacy is not known.

Prognostic scoring systems have been developed in order to inform clinician and patient decision-making processes around MPEs [4,12], and an online decision support tool is now also available [13]. However, with little known research into the psychosocial impact of living with and managing an IPC, the information underpinning such decision-making is incomplete.

The British Thoracic Society (BTS) draft guidelines [10] advocate for patients and/or their relatives to be supported to complete their own drainage, promoting independence and self-management. Studies have shown that patient empowerment increases patient satisfaction, adherence to treatment plans, and care outcomes [14]. This has increasingly been applied to cancer as survival times lengthen, but the suitability of this approach to later-stage cancer, MPE, and the use of IPC has not been evidenced.

Pleural services are an emerging specialty, the demand for which is only likely to increase [3-4]. Evidence is required to better design IPC care provision, including understanding the psychosocial impact of implanting an IPC, knowledge of patient support needs, and an appreciation of patient and nurse attitudes towards self-management. Little is known about the extent of the literature on this subject. Given the broad field of inquiry, and in order to permit flexibility, a scoping review was selected as an appropriate methodology to address this knowledge gap.

## Review

Study design

The primary objective of the review was to identify research into the psychosocial impact of IPC, either from the patient's perspective or that of clinical staff or family/unpaid carers. Initially, we limited study inclusion to qualitative research; however, in response to peer review, this was widened to include quantitative methods as well. A secondary objective was to capture reports on patient support needs or attitudes towards self-management of IPC. As a scoping review, the intention was to report on the extent, range, and nature of the studies identified before providing a summary of findings. In the event of a sufficient number of appropriate studies being identified, evidence synthesis was planned; for qualitative data, this was to be an interpretive analysis and narrative synthesis with line-by-line coding to facilitate the identification and mapping of themes.

Quality of life studies were deemed relevant to the primary research question in so far as they may capture elements of psychosocial impact. However, looking at (sometimes aggregated) numerical scores from QoL, dyspnoea, or performance scale questionnaires provides little depth or insight into the psychosocial impact IPC may have upon individual patients. Moreover, a 2019 systematic review of HRQoL in patients with IPC for MPE (as measured by quantitative tools) showed inconsistent results [11]. In order to focus solely on the psychosocial aspects of QoL, explore these in depth, and capture patient perspectives and experiences, we limited the inclusion of QoL studies to those employing a qualitative methodology.

A review protocol was developed in line with the Preferred Reporting Items for Systematic Review and Meta-Analyses (Prisma) Scoping Review (ScR) guidelines [15], published on the Centre of Open Science OSF platform [16], and subsequently revised in line with peer review.

Search Strategy

A search strategy was developed with reference to the 'Sample, Phenomenon of Interest, Design, Evaluation, and Research Type (SPIDER) framework [17] (Table 1). The phenomenon of interest was the IPC; patients, their families and/or informal carers, and healthcare workers were the target sample group. Given the breadth of this group, this step was omitted from the search strategy.

**Table 1:** Search strategy

	Variable	Search terms	Synonyms and other related search terms
Sample	Patients, healthcare workers, family and/or unpaid carers	Given the non-specificity of the sample this was omitted from the search string	
Phenomenon of interest	Insertion and management of an indwelling pleural catheter	Indwelling pleural catheter	Pleural catheter
Design	Any study design		
Evaluation	Psychosocial impact of indwelling pleural catheter insertion and management in the community	Psychosocial	Activities of daily living, activities of daily life, quality of life, happiness, control, psychological or social adaptation or adjustment, social behaviour, social stigma, impact
Research type	Any research type		

A search string was compiled based on the above strategy, utilizing keywords, and amended for individual database use. For nursing databases, fewer terms were used; for example, "Indwelling pleural catheter*" (anywhere in text) returned only 25 hits on the British Nursing Index (BNI), and so no further refinement was required.

The strategy was modified as required for individual databases and implemented in the following online databases between October 24 and October 28, 2022: BNI, Cumulative Index of Nursing and Allied Health Literature (CINAHL), Cochrane, Excerpta Medica Database (Embase), Exerpta Medica Care (Emcare), E-thesis Online Service (EThOS), Medical Literature Analysis and Retrieval System Online (Medline), National Grey Literature Collection, Psychological Information Database (PsycInfo), and PubMed.

The search string adapted for use in CINAHL is provided below as an example: "Indwelling pleural catheter*" AND ((impact*) OR (effect*) OR (experience*) OR (psychological*) OR (psychosocial) OR (social) OR ("activit* of daily li*") OR (behaviour) OR (stigma) OR ("quality of life")).

In addition, the reference lists of systematic reviews were searched for relevant papers, and a search of PubMed was conducted using controlled vocabulary headings (MeSH terms). The pleural effusion heading encompassed sub-headings of nursing, psychology, and complications.

((pleural effusion[MeSH Terms]) AND (catheters, indwelling[MeSH Terms])) AND ((life change events[MeSH Terms]) OR (life experiences[MeSH Terms]) OR (psychological adaptation[MeSH Terms]) OR (psychological adjustment[MeSH Terms]) OR (social adaptation[MeSH Terms]) OR (social adjustment[MeSH Terms]) OR (social behaviour[MeSH Terms]) OR (activities of daily living[MeSH Terms])).

Eligibility Criteria

For the primary objective, the following criteria were applied to the selection of literature for inclusion.

Inclusion criteria: Study of patients with an IPC and their care; study evaluating the psychosocial impact of an IPC on patients and/or family/unpaid carers; study reporting on the quality of life (where assessed by qualitative methods); IPC inserted for the management of pleural effusion (whether malignant or non-malignant, including empyema, and irrespective of aetiology).

Exclusion criteria: Published in a language other than English; no mention of the quality of life or psychosocial outcomes in the title or abstract; quality of life measured quantitatively; not primary research.

**Figure 1:**
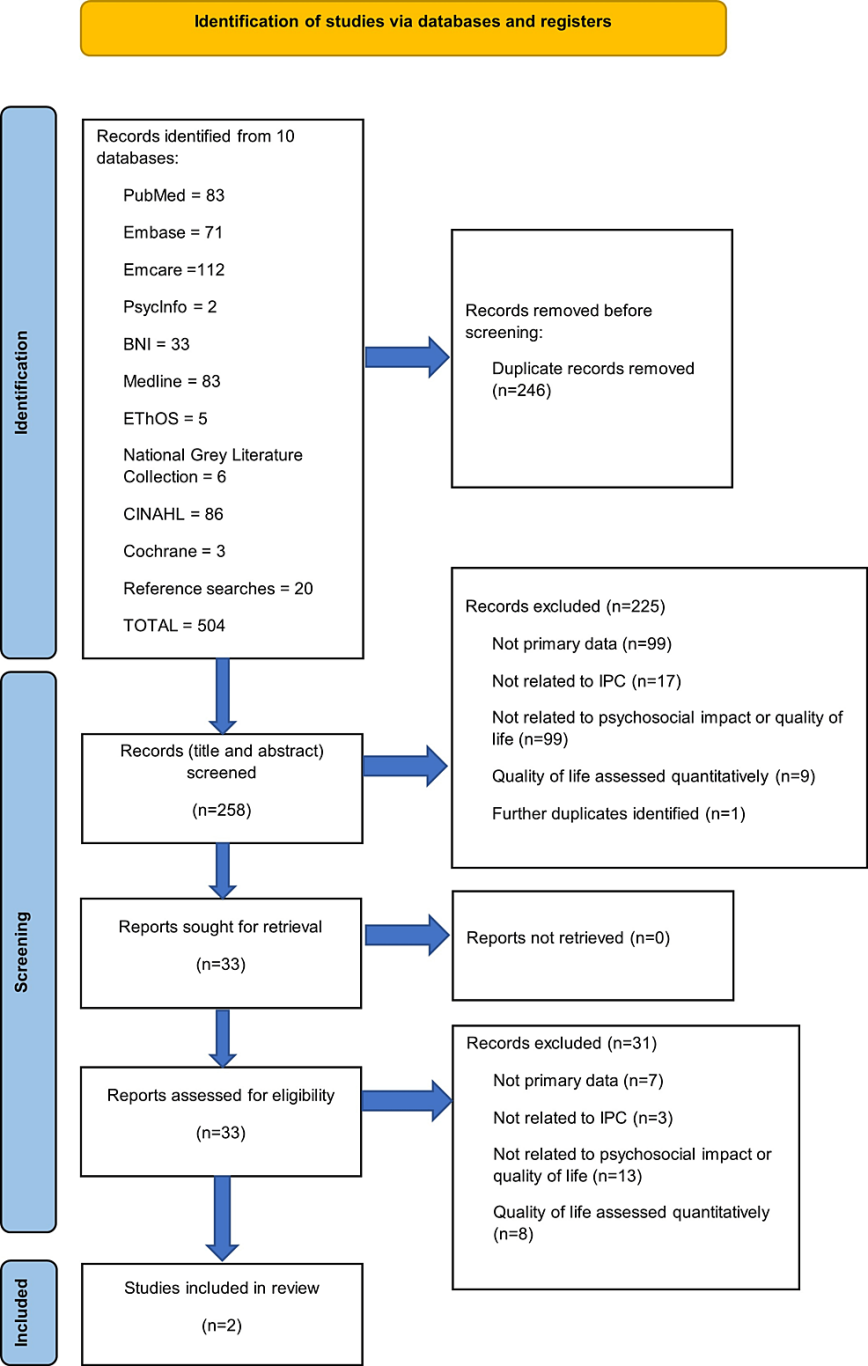
PRISMA flow diagram BNI: British Nursing Index, CINAHL: Cumulative Index to Nursing and Allied Health Literature, Embase: Excerpta Medica Database, Emcare: Exerpta Medica Care, EThOS: E-thesis Online Service, Medline: Medical Literature Analysis and Retrieval System Online, PsycInfo: Psychological Information Database, and PubMed.

Articles were screened for inclusion using the above criteria (Figure 1, PRISMA flow diagram). Inclusion criteria pertaining to the secondary objectives were limited to the English language, primary data, and the study of IPC.

Results

Two studies met the inclusion criteria (Table 2). Seventeen studies were identified due to ‘quality of life’ appearing in the abstract; however, these were quantitative in nature (typically standardized QoL questionnaires compared across treatment arms or pre- and post-insertion). Interrogation of the questionnaire results by domain was rarely reported, and there was a lack of studies with qualitative methodologies. For the two studies included [18-19], it was not possible to assess the rigour of the methods used due to a lack of reporting. Similarly, the coherence of the reported findings could not be assessed due to a lack of analytical depth.

**Table 2:** Included studies References: Bielsa et al. [18], Muhammad et al. [19]

	Bielsa et al. (2014)	Muhammad et al. (2020)
Title	Indwelling pleural catheter for ambulatory care in patients with malignant pleural effusion	Indwelling pleural catheter for outpatient management of tuberculous empyema
Format	Conference abstract	Case study
Topic	Patient and caregiver satisfaction	Personal impact statement
Method	Telephone interviews	Not documented
Participant number	8	1
Participant type	Patients	Patient
Participant age	Mean 67 years (standard deviation 13)	26
Participant- gender	6 male 2 female	Male
Aetiology	Malignant pleural effusion	Tuberculosis
Results	Improved Spitzer quality-of-life score: mean score before IPC = 4.6 (standard deviation 0.7); mean score post-IPC insertion = 6.6 (standard deviation 1.8). 4 care givers accepted IPC management and reported feeling safe, satisfied and well supported. 4 care givers reported lacking confidence to manage the IPC.	Endorsement of outpatient treatment; statement of improved ease of living.

Quality Assessment and Narrative Review 

It was planned to use the Critical Appraisal Skills Programme (CASP) checklist [20] for appraising study quality. However, given the identification of only two studies (one of which was not published in full and the other which was only tangentially relevant), this was deemed unpracticable. Instead, the studies, along with their methodological limitations, are described below. It should be made clear that these are not criticisms of the research per se, but rather that the research questions were not fully aligned with the purpose of this review and the format of the publications did not allow for full exposition.

"Indwelling pleural catheter for ambulatory care in patients with malignant pleural effusion," the study by Bielsa et al. [18]: This study was presented as a conference abstract; it does not appear to have been published in full elsewhere, and it did not prove possible to contact the authors for further details. The study was conducted in Spain with the aim of assessing patient and caregiver satisfaction with IPC at a single centre. No funding or competing interests were declared. The abstract reports that eight consecutive patients with IPC inserted for MPE were recruited and interviewed by telephone. The timeframe between IPC insertion and the interview is not described. Quality of life was assessed quantitatively, and the median before- and after-IPC insertion scores were reported. The number of informal family caregivers who had taken on the role of IPC management was four (50%); they were reported to have felt well-informed, satisfied, and safe; the other four did not feel sufficiently confident to manage the IPC. The participant numbers are small, with no discussion of data power or saturation; the depth and format of the interviews are unclear; what is reported is largely quantitative, with a summary of caregivers feelings about IPC management. How the summary was arrived at from the interviews is not reported, nor are the structure and questions used in the interviews. The reasons for caregivers feeling safe or lacking confidence were either not explored or not reported.

"Indwelling pleural catheter for outpatient management of tuberculous empyema," the study by Muhammed et al. [19]: the hierarchy of evidence proposed by Evans [21] rates case studies as poor for the evaluation of the effectiveness, appropriateness, and/or feasibility of healthcare interventions. In this UK case study, the majority of the article pertains to medical history, treatment, and clinical outcomes. No funding or competing interests were declared for this study. The first-person experience is limited to a single, four-sentence ‘patient experience’ statement. This does, however, convey very clearly the frustration the patient felt at prolonged hospitalization and the resulting concerns he had about the security of his employment. The individual stated that the enablement of outpatient treatment was one of his prime motivators for accepting the IPC and that its implantation allowed him to return to work and made his life much easier. Inherent in the case study format is the selection of a noteworthy or atypical patient; in this case, the IPC was inserted for tuberculous empyema rather than MPE, and his prior hospital treatment had been on an inpatient basis for an uncharacteristically long 80 days. This may have had some bearing on the emphasis placed on outpatient treatment in his statement. It is not made clear whether the patient was undertaking the drainage themselves or whether the IPC was being managed by healthcare professionals.

Excluded Studies

The simple lack of studies meeting the inclusion criteria answers the stated aim of this scoping review, highlighting the absence of research investigating the psychosocial impact of IPC or reporting on the quality of life using qualitative methods. However, given this finding, it deemed useful to present the more relevant of the excluded studies, an overview of which might add value to anyone working or researching in this field (Table 3).

**Table 3:** Excluded studies of relevance References: Aboudara et al. [22], Huisman‐de Waal et al. [23], Sivakumar [24], Thomson et al. [25]. IPC: indwelling pleural catheter, MPE: malignant pleural effusion, QoL: quality of life (QoL).

	Aboudara et al. (2021)	Huisman‐de Waal et al. (2011)	Sivakumar (2021)	Thomson et al. (2013)
Title	A survey-based study of patient-centered costs associated with indwelling pleural catheters	‘High-tech’ home care: overview of professional care in patients on home parenteral nutrition and implications for nursing care	Interventions to improve health-related quality of life in malignant pleural effusion	The psychosocial impact of home use medical devices on the lives of older people: a qualitative study
Format	Journal article	Journal article	PhD thesis	Journal article
Country	United States of America	Netherlands	United Kingdom	United Kingdom
Topic	Socio-economic impact	Nursing care; implantable device	Quality of life	Use of home medical devices
Method	Cross-sectional survey	Questionnaire and interview	Patient and public involvement group; unstructured discussion	Semi-structured interviews of patients and partners
Reason for exclusion	Not qualitative	Not IPC	Relates to MPE but not IPC specifically	Not specific to implanted medical devices or IPC
Relevance	Reports quality of life issues in more detail than standard validated QoL questionnaires	Implantable device and its nursing care	Research relates to MPE QoL	Researches the psychosocial impact of medical devices in the home in an older population, including 2 participants with implanted devices
Participant number	20	64 patients (questionnaire) 17 nurses (interview)	7	12
Participant type	Patients with an IPC	Patients in receipt of parenteral nutrition. Nurse specialists or responsible homecare nurses.	Patients with an MPE n=5; family/informal caregivers n=2	Aged over 65 and using a medical device in the home patients n=12; partners n=7
Participant age	Median 64 years (interquartile range 58-71)	Mean 53 years (standard deviation 14.7; range 18-77)	Not reported	Mean 72 (range 65-83)
Participant- gender	13 female 7 male	42 female 22 male	Not reported	10 female 9 male
Aetiology	Malignant pleural effusion (in 19 of the 20)	Not applicable	Malignant pleural effusion	Not applicable
Results	No participant reported missing an important event due to their IPC, nor having insufficient money due to their IPC. 30% said there were activities they could not do any more, half of whom said this impacted negatively on their wellbeing and quality of life. 45% reported not travelling somewhere due to their IPC. 85% reported receiving care from an informal/unpaid care giver. No informal carers had to give up work but 12% were reported to have taken time off work to perform these duties.	Questionnaire revealed the most frequently reported problems were under the social behaviour, psychic autonomy, communication, and emotional stability subscales. In only 4% of outpatient clinic appointments were psychosocial problems such as anxiety, depression and coping discussed. In other contacts (e.g., telephone consultation) psychosocial counselling was not documented but nurses reported this typically occurred. Psychosocial issues were reported as the most important issue discussed at 4 out of the 5 home visits analysed.	Asked what factors of care were important to patients with MPE, five areas were reported to have particular importance to quality of life – (1) symptoms (and symptom management); (2) satisfaction with medical care; (3) independence; (4) cognitive function; and (5) mental wellbeing.	Two main themes identified: (1) Striving to maintain self-esteem. This included sub-themes of feeling powerless (particularly around the decision to accept the device), personal control (empowerment), mastering the device, and comparing oneself to others (with same condition/device). (2) The Social device. Identification of different ways in which devices influence social interactions which were grouped into ‘bringing people together’ and ‘disrupting social harmony’. The former included notions of joint ownership and co-management; while the latter included creating barriers within couples and in wider social contexts.

Discussion

The primary objective of the review was to identify and report on the extent, range, and nature of research into the psychosocial impact of indwelling pleural catheters. Due to the inclusion of QoL in the search and the difficulty of limiting this aspect of the search to qualitative methodologies, the search returned a large number of studies. With only two studies meeting the inclusion criteria and these containing little depth of reporting, it was not possible to identify emerging themes. From this, it can be concluded that there has been little to no research into the psychosocial impact of IPC. However, secondary objectives were to capture any reports on patient support needs or attitudes towards self-management. During the screening process, several studies were identified that failed to meet the inclusion criteria but were relevant to these objectives.

Self-Management

The term ‘self-management’ - in the context of IPC - tends to be used to differentiate between management by a healthcare professional and management by the patient. However, this usage has the capacity to underserve family/unpaid carers whose involvement it is important to recognize. Aboudara et al. [22] state that many US clinicians assume that patients will be draining the fluid themselves, but in practical terms, the siting of the IPC and length of tubing may make it difficult for some patients to truly self-manage, and management by a patient-family caregiver dyad is perhaps more common. Aboudara et al. [22] report that patients rely heavily on members of their social network, with 85% of respondents receiving care from an informal/unpaid caregiver; such carers are an understudied population (in relation to IPC) and little is known about their own support needs. Moreover, the reasons for this high incidence of family/carer involvement need to be established and may be an important consideration when discussing the possible insertion of an IPC, particularly given the length of time for which such support may be needed (Asciak et al. [26] report a mean survival time from IPC insertion of 324 days (non-MPE group) and 214 days (MPE group)). Aboudara et al. [22] suggest that discussions about the role of caregivers should become a routine part of the consent process.

Thomson et al. [25] studied the home use of non-implantable medical devices in older patients and identified ways in which devices influence social interactions. These included both bringing people together (through notions of joint ownership and co-management) and creating barriers (either within couples or in wider social contexts). An understanding of how this might apply in the context of IPC might help patients and families navigate this territory and inform discussions around IPC management.

Bielsa et al. [18] reported that the willingness of family caregivers to undertake IPC care - and to feel confident and safe in doing so - varied. This variability suggests people’s experiences of living with and managing their IPC may be quite different. The reasons underlying this variance are not yet fully understood. Future work could explore these attitudes with a view to developing appropriate support for those who wish to consider self-management.

Finally, with respect to self-management, it is important to define the term and whether it is being used to indicate actual self-care (management by the patient themselves/alone) or being used in a more expansive sense.

Empowerment

Thomson et al. [25] identified the maintenance of self-esteem as important in counteracting the potential negative psychological impacts of disease and having to use a home medical device, describing sub-themes of empowerment/personal control, and mastery of the device. Participants reported enhanced feelings of control over their illness as a result of using home medical devices and bolstered self-esteem as a result of their perceived mastery of the equipment. This study included older people managing non-implanted medical devices at home; the extent to which these findings might be applicable to IPC is not clear. However, Muruganandan et al. [27] in their IPC study found that patients in the aggressive (more frequent) drainage group reported better scores in the EQ-5D-5L health-related quality of life (HRQoL) questionnaire, despite reporting no better outcomes in terms of shortness of breath or pain. Murungandan et al. [27] hypothesize that the improved HRQoL score might be the result of patients feeling an increased sense of control (due to the frequent drainage) in the face of their underlying condition. Whether IPC self-management (irrespective of drainage frequency) can give a similar sense of empowerment and whether this enhances HRQoL scores is not known; however, self-management might facilitate aggressive drainage plans (where desirable) by alleviating demand on healthcare staff and removing the need for patients to stay home for visiting healthcare professionals, thereby increasing the acceptability of the intervention.

Independence

Shafiq et al. [28] describe IPC as 'a patient-centred intervention that enables the patient and their caregiver to self-determine the desired frequency and volume of drainage, besides affording the comfort of at-home drainage without hospitalization or visits to a health care provider' (p.747). The individual featured in the case study by Muhammed et al. [19] stated that the enablement of outpatient treatment was one of the prime motivators for his accepting the IPC and that its implantation allowed him to return to work and made his life much easier. Similarly, independence was identified as an important factor of care (in respect of quality of life) in a study of patients with MPE [24].

A 2019 systematic review of interventions for MPE showed improved HRQoL scores with IPC [11], but the results were described as modest and inconsistent, and the overall quality of the included studies was inadequate. A more recent study by Sivakumar [24] found statistically significant improvements in the ‘role’ domain of the European Organization for Research and Treatment of Cancer (EORTC) QoL questionnaire after IPC insertion; specifically, an improvement in work, activities of daily living, and leisure activity limitations. The cross-sectional survey identified in our literature search [22] conflicts with this; 30% of their respondents said there were activities they could not do anymore, half of whom said this impacted negatively on their well-being and quality of life. This finding surprised the authors, who were unable to explore it further due to the study methodology. The extent to which IPC impacts this aspect of QoL may depend on the individual activities of that patient and the value they place on them. In light of this, and despite the generally reassuring findings on the positive impact of IPC on HRQoL, Aboudara et al. [22] argue that the potential impact on activities such as swimming and travel should form an essential part of the discussions taking place prior to IPC insertion, stating 'IPC may pose a much greater socio-economic burden on certain patients and their caregivers than realized by clinicians' (p.365). They advocate for a tailored approach based on patients’ caregiver networks and life priorities. This is echoed by findings from Grindall et al. [13], who report that the main influences on people’s treatment decisions (for MPE) were personal aspects of their lives, including how active or not they were and what support they had available at home. Messeder et al. [29] note that some patients dislike a semi-permanent drain under their clothing, suggesting that inconvenience, hygiene, and concerns around infection might play a role. In particular, they suggest that those patients with a good prognosis who follow an active lifestyle may prefer to avoid an IPC. In summary, although the literature as a whole is reassuring about improved QoL following IPC insertion, this may not be universal and, in terms of facilitating independence, might depend on previous health and lifestyle.

Psychological Impact

In Sivakumar’s study [24] of patients with an MPE (but not necessarily an IPC), independence and mental well-being were both identified as factors of care that were particularly important to their quality of life. In the study of parenteral nutrition by Huisman‐de Waal et al. [23], the most frequently reported problems were under the social behaviour, psychic autonomy, communication, and emotional stability categories. Interestingly, they found that in only 4% of outpatient clinic appointments were psychosocial issues (such as anxiety, depression, and coping) discussed, yet at home visits these were reported as the most important issues discussed. There are important differences between the indwelling medical devices required for the delivery of parenteral nutrition and IPC; there are also important differences in the patient group and underlying disease processes. The extent to which IPC may impact psychosocial well-being, the extent to which this is discussed with health professionals, and the extent to which these needs are supported by current IPC services are not known. It is therefore important that effective ways of assessing impact are developed. Sivakumar et al. [11] argue that qualitative research is required to characterize psychosocial outcomes and that this could be tied to the development of a disease-specific instrument for measuring HRQoL in MPE populations. They point out that psychological impact is likely to be mediated through patient expectations as well as social and cultural background. In addition to developing impact assessments, a greater understanding of the range of potential IPC impacts should enable patients to make informed treatment decisions specific to their personal circumstances.

Healthcare Burden

Saqib et al. [30] state that improving QoL is among the most important management goals and that both avoiding hospital admissions and reducing the number - and length - of hospital stays may be considered constituent parts of QoL. A systematic review and meta-analysis by Iyer et al. [31] reported a shorter length of total hospital stay and a lower number of repeat pleural interventions in those with an IPC when compared to pleurodesis for MPE. For those patients with a terminal diagnosis, how many of their remaining days are spent in the hospital may be a very important consideration. Fortin and Tremblay [9] conclude that "while pleurodesis approaches are associated with a shorter initial treatment phase, more rapid pleurodesis, and the absence of the need for chronic catheter care, they may also be associated with lower rates of effusion control and an increased need for repeat pleural intervention" (p. 1054). Asciak et al. [26], however, argue that when comparing IPC to other interventions, the majority of studies have focused on short-term outcomes (such as hospital stays), which do not capture the full patient and healthcare-associated impact of IPC. They found 17% of MPE patients required an additional IPC-related review (e.g., for ultrasound assessment), and of these, 23% required further review, a burden that may not be captured in study reporting. Moreover, they point out that their study did not capture data on GP or community nurse contact, phone contact with pleural services, or the patient impact or healthcare resources involved in home drainage. Both Asciak et al. [26] and Sivakumar et al. [11] suggest that the data captured tends to be from the hospital perspective (for example, time-until-no-further-drainage) rather than the patient perspective (for example, the impact of the three-times-a-week drainage) or community nurse visit data. They argue that the impact of IPC on both patients and community services may therefore be underestimated. From a health economics perspective, Fontin and Tremblay [9] state that IPC appears to be a cost-effective strategy compared with others until community nursing care (including dressings) of two hours or more is required for IPC drainage on a weekly basis, a finding supported by Dipper et al. [32]. This is roughly equivalent to draining three times a week, a not uncommon regime in the initial stages of IPC management, and so cost advantages over the life of the IPC may be equivocal.

Self-Care and IPC Infection

IPC infection rates are typically low; Fysh et al. [33] report that IPC-related pleural infections occurred in less than 5% of over 1,000 Australian patients, and pleural infections may usually be controlled with antibiotics [34]. However, the systematic review by Iyer et al. [31] identified an increased risk of cellulitis when compared to pleurodesis in patients with MPE; they point out that this is likely to vary in different clinical contexts, influenced by local practises and populations.

Akram et al. [35] identified IPC domiciliary care education as a statistically significant independent risk factor in a multivariate analysis, reducing the risk of IPC infection (adjusted odds ratio [AOR] 0.18; 95% confidence interval [CI; 0.05-0.66]). The study examined IPC infection rates in Pakistan, where 28 participants had domiciliary IPC education regarding management at home (including aseptic technique). The generalizability of these findings is uncertain as the infection rate is unusually high (26%) and it is unclear whether the comparator group - those who did not receive domiciliary IPC education - were managing their own drainage or if this was being done by community healthcare professionals.

Limitations

The studies were assessed against the inclusion criteria by one researcher only; it is possible that a second reviewer might have made different decisions, but given the simplicity of the inclusion criteria, this was deemed unlikely. The two studies identified were comprised of a conference abstract (not published in full elsewhere) and a case study with limited qualitative reporting. Given the limitations inherent in the case study format and the lack of reporting depth possible in a conference abstract, it was neither possible nor worthwhile to undertake a quality assessment. The discussion of papers that did not meet the inclusion criteria - and whose relevance was judged by the individual researcher without strict inclusion criteria - creates the potential for selection bias; however, this literature is not presented as comprehensive and serves only to stimulate discussion. There appears to be little literature on the self-management of IPC and whether this has any relationship to infection rates; however, the literature search was not directed specifically at this outcome, so this cannot be stated with confidence.

Research Implications

There is a need to develop research to better understand and characterize the psychosocial impact of IPC insertion and management. The support needs of patients and family/unpaid carers require assessment, and appropriate tools need evaluation. The meaning of the term self-management needs expounding when used in studies to clarify whether this refers only to the patient or includes other family/unpaid carers. The limited research on self-management suggests that family/unpaid carer involvement is common, but the extent of involvement is variable. Research to better understand attitudes towards and the nature of self-management is needed.

## Conclusions

We have presented a scoping review of the literature relating to the psychosocial impact of IPC, patient support needs, and attitudes towards self-management. IPCs are designed to improve quality of life - to improve pain and dyspnoea - but we know little about the other ways in which they may affect patients’ lives. Looking at scores on HRQoL tools or dyspnoea scales - while undoubtedly useful - tells us little about the psychosocial impact these interventions may have upon individual patients, nor do they give any insight into mechanisms for the amelioration of such unintended effects. While there is a dearth of literature on the psychosocial impact of IPC, an acknowledgement of the potential for impact and the need to consider this when discussing IPC insertion is a common theme. The importance of family or informal/unpaid carers in IPC management was identified by one study; the impact this may have on social relationships has been researched in other fields but not in relation to IPC.
